# Association between drinking patterns and diabetic kidney disease in United States adults: a cross-sectional study based on data from NHANES 1999–2016

**DOI:** 10.1080/0886022X.2025.2454970

**Published:** 2025-01-22

**Authors:** Xusheng Yang

**Affiliations:** Department of Nephrology, Beijing Rehabilitation Hospital, Affiliated to Capital Medical University, Beijing, China

**Keywords:** Drinking pattern, logistic regression, diabetes mellitus, diabetic kidney disease, NHANES

## Abstract

**Objective:**

This cross-sectional study aimed to investigate the association between drinking patterns and prevalence of diabetic kidney disease (DKD) among adults in the United States.

**Methods:**

Data were analyzed from the NHANES surveys conducted between 1999 and 2016, including 26,473 participants. Drinking patterns were categorized by frequency (weekly, monthly, or yearly) and quantity (light, moderate, or heavy, based on daily consumption). Among participants with diabetes, DKD was defined using the albumin-to-creatinine ratio (ACR ≥30 mg/g) and estimated glomerular filtration rate (eGFR <60 mL/min/1.73 m^2^). Multivariable logistic regression models were used to evaluate associations, adjusting for potential confounders across the four models. Subgroup analyses were performed to assess the effects of modification by age, sex, race, BMI.

**Results:**

Drinking patterns and DKD were analyzed among 26,473 US adults (mean age, 46.6 years; 53.7% male). After adjusting for multiple confounders, heavy alcohol consumption was associated with a higher risk of DKD than light drinking (OR = 1.23, 95% CI, 1.04–1.46; *p* = 0.016). Conversely, moderate drinking frequency (3–4 days per week, 2–5 days per month, 3–126 days per year) was associated with a reduced DKD risk (OR = 0.67, 95% CI, 0.49–0.91; OR = 0.75, 95% CI, 0.56–0.99, OR = 0.71, 95% CI, 0.58–0.86, respectively). A nonlinear association was observed between alcohol consumption frequency and DKD in terms of weekly and yearly drinking days.

**Conclusion:**

This study highlights the importance of drinking behavior in the management of diabetic kidney disease. Daily alcohol consumption was associated with an increased risk of DKD, whereas moderate alcohol consumption was associated with a reduced risk. These findings suggest that moderate drinking frequency may not exacerbate renal burden in individuals with diabetes and provide new perspectives for clinical interventions.

## Introduction

Diabetic kidney disease (DKD) is a prevalent microvascular complication of diabetes with a rising incidence worldwide. DKD is strongly associated with premature mortality [1], highlighting the urgent need for effective preventive and management strategies. Despite their overlapping characteristics, DKD differs from chronic kidney disease (CKD) owing to its unique pathophysiological mechanisms, including hyperglycemia, insulin resistance, and advanced glycation end-products [2]. These distinctions necessitate DKD-specific research as findings from CKD studies may not fully translate to DKD.

Alcohol consumption is common worldwide, with nearly half of the U.S. population aged 12 or older reporting regular intake [3]. Studies [4–8] on CKD have suggested that moderate alcohol consumption may confer cardiovascular benefits, potentially reducing the risk of CKD, whereas heavy alcohol consumption may exacerbate oxidative stress, hypertension, and kidney damage. However, findings remain inconsistent, and little is known about the role of alcohol consumption in DKD, a condition uniquely influenced by hyperglycemia and diabetes-related comorbidities [9].

This study aimed to explore the relationship between alcohol consumption patterns and DKD risk among adults in the United States using data from the National Health and Nutrition Examination Survey (NHANES). By analyzing data from 26,473 participants, this study sought to provide new insights into the influence of alcohol consumption behaviors on DKD risk, providing strategies to improve the prevention and management of this diabetic complication.

## Methods

### Database

This cross-sectional study relied on data from the NHANES, a nationwide health survey conducted by the National Center for Health Statistics (NCHS) in the U.S. NHANES employs a multistage, stratified, probability sampling design to ensure nationally representative data for the non-institutionalized U.S. population. Data collection included household interviews, physical examinations at mobile examination centers (MECs), and laboratory tests conducted by qualified medical professionals. Additional information can be found at (https://www.cdc.gov/nchs/nhanes/about_nhanes.htm). The original study was approved by the NCHS Ethics Review Board and all participants provided written informed consent.

### Study population

**Figure 1.**
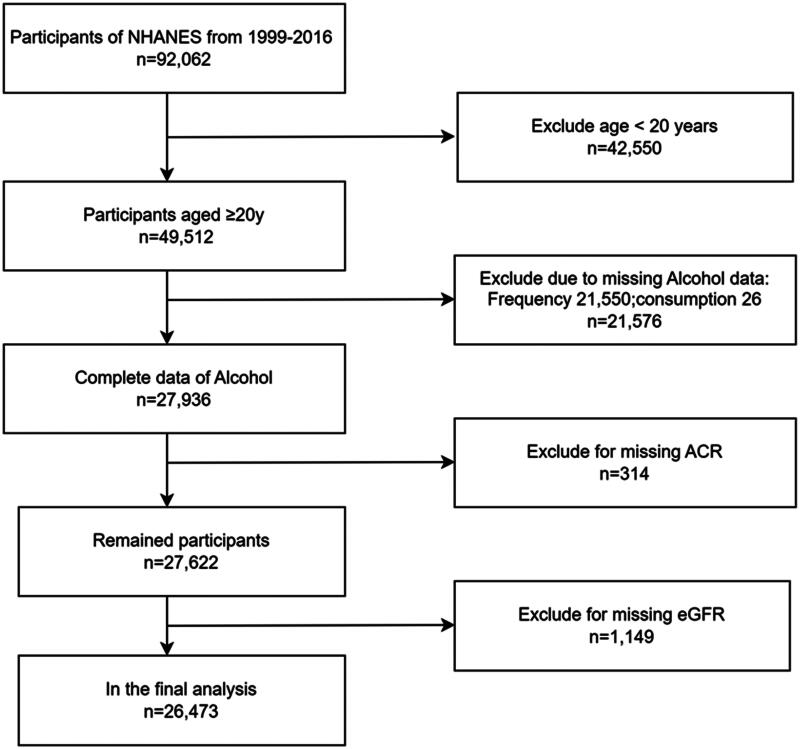
Flowchart of the participants included.

We restricted our analysis to participants aged 20 years or older. The exclusion criteria were: missing data on daily alcohol consumption or drinking frequency; missing data for key kidney function markers, including urinary albumin-to-creatinine ratio (ACR) and estimated glomerular filtration rate (eGFR); and participants younger than 20 years of age. Of the 92,062 initial participants, we excluded 42,550 participants younger than 20 years, 21,576 with missing daily alcohol consumption or drinking frequency data, 314 individuals with missing ACR measurements, and 1,149 with missing eGFR data, resulting in a final sample of 26,473 participants. The participant selection process is illustrated in Figure 1.

At the MEC, trained interviewers utilized the Computer-Assisted Personal Interviewing (CAPI) system to inquire about alcohol consumption habits, including the frequency of drinking per week, month, and year as well as the quantity consumed. A drink was defined as a 12 oz. beer, a 4 oz. glass of wine or an ounce of liquor (for more information about the NHANES, see www.cdc.gov/nchs/nhanes.htm). Alcohol intake was divided into three levels, namely: current light drinker (an average of ≤ 1 drink per day for females, ≤ 2 drinks per day for males over the past year, or binge drinking [≥ 4 drinks/occasion for females, ≥ 5 drinks/occasion for males] on 1 day per month); current moderate drinker (≤ 2 drinks per day for females, ≤ 3 drinks per day for males, or binge drinking on 2–5 days per month); current heavy drinker (≥ 3 drinks per day for females, ≥ 4 drinks per day for males, or binge drinking ≥ 5 days per month) [10]. According to the NHANES alcohol questionnaire items ALQ120Q (How often did you drink alcohol over the past 12 months) and ALQ120U (number of days drank alcohol per week, month, year), alcohol consumption frequency is categorized into weekly, monthly, and yearly intervals based on days.

### Diabetic kidney disease

Diabetes mellitus (DM) was defined as (1) a previously reported diagnosis by medical professionals, (2) fasting plasma glucose ≥7.0 mmol/L, (3) glycosylated hemoglobin (HbA1c) ≥6.5 %, or (4) taking diabetes drugs. The urine albumin/creatinine ratio was used to calculate the ACR. The eGFR was calculated using the formula eGFR = 142 × min(SCr/k, 1)^a * max(SCr/k, 1)^(–1.200) * 0.9938^Age * 1.012 [if female] [11]. ACR ≥30 mg/g or eGFR < 60 mL/min/1.73 m^2^ was used to diagnose DKD in DM patients [12].

### Study covariates

Data collected included age, sex, and race/ethnicity, which were categorized into five groups: Mexican American, other Hispanic, non-Hispanic White, non-Hispanic Black, and Others. Body Mass Index (BMI) was calculated using weight and height (kg/m^2^) and classified according to the previous literature categories [13]: Normal weight (<25), Overweight and Obese (≥25). The smoking status was classified as daily, some days, or not at all. Milk consumption was categorized as never, rarely (less than once a week), sometimes (once a week or more, less than once a day), often (once a day, or more), and varied. Diagnoses of hypertension, coronary heart disease, heart failure, stroke, and liver disease were based on medical history as reported by healthcare providers.

### Statistical analysis

All continuous variables adhering to a normal distribution were presented as mean ± standard deviation, while categorical variables were expressed as frequencies (%). Group comparisons of continuous variables were performed using either the independent samples *t*-test or the Mann-Whitney U-test, depending on distribution normality, while categorical data were compared using the chi-square test or Fisher’s exact test, as appropriate.

Logistic regression analysis was used to investigate the relationship between drinking patterns (quantity and frequency of alcohol consumption) and DKD. The frequency of alcohol consumption was treated as a categorical variable. Covariates were selected based on clinical relevance, existing literature, and significant variables identified in univariate analysis. Four models were constructed: Crude model was adjusted for none. Model 1 was adjusted for age, sex, and race. Model 2 was additionally adjusted for BMI, education level, milk consumption, and current smoking status. Model 3 was further adjusted for history of heart failure, coronary heart disease, stroke, liver disease, and hypertension.

Trend tests were conducted using the median values of alcohol consumption and frequency quartiles as continuous variables. In the case of non-linear correlations, a two-piecewise regression model was used to determine the threshold effect of alcohol consumption quantity and frequency on DKD. Predefined subgroup analyses were performed based on the subgroup variables.

In the Statistical Analysis subsection of the Methods, we observed that missing data constituted less than 5% of the dataset. To handle these missing data properly, we implemented a multiple imputation strategy. Multiple imputations were performed using a Fully Conditional Specification (FCS) implemented by the MICE algorithm, as described by Van Buuren and Groothuis-Oudshoorn [14]. Each variable had its own imputation model. Built-in imputation models are provided for continuous (predictive mean matching, normal), binary (logistic regression), unordered categorical (polytomous logistic regression), and ordered categorical (proportional odds model) data. MICE can also be used to impute continuous two-level data (normal model, pan, and second-level variables). Passive imputation can be used to maintain the consistency between variables. Various diagnostic plots are available to inspect the quality of imputations. The analyses were repeated using the complete data set for comparison. Sensitivity analyses were conducted to assess the robustness of the findings and the potential impacts of the different association inference models. The effect sizes and p-values from all models were reported and compared.

All analyses were performed using Free Statistics software version 1.9.2. *p* < 0.05 (two-sided) was considered statistically significant.

Free Statistics is a software package that provides intuitive interfaces for the most common analyses and data visualizations. Free Statistics uses R as the underlying statistical engine, and the graphical user interface (GUI) is written in Python. Most analyses can be performed with only a few clicks. It was designed for reproducible analysis and interactive computing (Free Clinical Medical Technology Co., Ltd., Beijing, China; http://www.clinicalscientists.cn/freestatistics/).

## Results

### Baseline characteristics of participants

Among the 49,512 adults aged 20 years or older, 21,550 were excluded because of missing data on drinking frequency, 26 because of missing data on alcohol consumption levels, and 1,463 because of missing kidney function data, leaving 26,473 participants for the analysis. The baseline characteristics of the included and excluded participants are shown in Supplementary Table 1. The mean age of the 26,473 participants was 46.6 years, and 53.7% were male. A total of 1,101 participants (4.2%) were classified as having diabetic kidney disease (DKD). The prevalence of DKD was higher (5.6%) among participants who reported a yearly drinking frequency. This group also had a greater proportion of individuals with a high school education or less (50.3%) and a higher prevalence of heart failure, coronary heart disease, and stroke (2.7%, 3.7%, and 3%, respectively) (Table 1).

**Table 1.** General characteristics according to alcohol consumption frequency.

Variables	Total (*n* = 26,473)	Alcohol Consumption Frequency			*P-value*
		Week (*n* = 10,978)	Month (*n* = 6,824)	Year (*n* = 8,671)	
Age, Mean ± SD	46.6 ± 17.3	47.8 ± 17.3	43.1 ± 16.8	47.9 ± 17.4	<0.001
Sex, *n* (%)					<0.001
Male	14,209 (53.7)	7,170 (65.3)	3,541 (51.9)	3,498 (40.3)	
Female	12,264 (46.3)	3,808 (34.7)	3,283 (48.1)	5,173 (59.7)	
Race, *n* (%)					<0.001
Mexican American	4,631 (17.5)	1,547 (14.1)	1,346 (19.7)	1,738 (20)	
Other Hispanic	2,068 (7.8)	672 (6.1)	626 (9.2)	770 (8.9)	
Non-Hispanic White	13,054 (49.3)	6,138 (55.9)	3,032 (44.4)	3,884 (44.8)	
Non-Hispanic Black	4,873 (18.4)	1,974 (18)	1,273 (18.7)	1,626 (18.8)	
Other Race	1,847 (7.0)	647 (5.9)	547 (8)	653 (7.5)	
BMI^a^, Mean ± SD	28.6 ± 6.5	27.6 ± 5.7	28.7 ± 6.7	29.8 ± 7.1	<0.001
Smoking, *n* (%)					<0.001
Everyday	10,146 (38.3)	4,159 (37.9)	2,550 (37.4)	3,437 (39.6)	
Sometimes	2,894 (10.9)	1,264 (11.5)	923 (13.5)	707 (8.2)	
Never	13,433 (50.7)	5,555 (50.6)	3,351 (49.1)	4,527 (52.2)	
Education, *n* (%)					<0.001
Below high school	5,831 (22.0)	2,202 (20.1)	1,373 (20.1)	2,256 (26)	
High school grad/GED^b^/Equivalent	5,961 (22.5)	2,400 (21.9)	1,456 (21.3)	2,105 (24.3)	
Above high school	14,681 (55.5)	6,376 (58.1)	3,995 (58.5)	4,310 (49.7)	
DKD^*c*^, n (%)					<0.001
No	25,372 (95.8)	10,599 (96.5)	6,585 (96.5)	8,188 (94.4)	
Yes	1,101 (4.2)	379 (3.5)	239 (3.5)	483 (5.6)	
HTN^*d*^, *n* (%)					<0.001
No	20,159 (76.1)	8,322 (75.8)	5,445 (79.8)	6,392 (73.7)	
Yes	6,314 (23.9)	2,656 (24.2)	1,379 (20.2)	2,279 (26.3)	
Milk, *n* (%)					<0.001
Never	4,333 (16.4)	2,050 (18.7)	1,023 (15)	1,260 (14.5)	
Rarely (less than once a week)	4,180 (15.8)	1,762 (16.1)	1,110 (16.3)	1,308 (15.1)	
Sometimes (once a week or more)	7,554 (28.5)	3,130 (28.5)	2,035 (29.8)	2,389 (27.6)	
Often (once a day or more)	10,303 (38.9)	3,992 (36.4)	2,635 (38.6)	3,676 (42.4)	
Varied	103 (0.4)	44 (0.4)	21 (0.3)	38 (0.4)	
Heart Failure, *n* (%)					<0.001
Yes	550 (2.1)	198 (1.8)	122 (1.8)	230 (2.7)	
No	25,923 (97.9)	10,780 (98.2)	6,702 (98.2)	8,441 (97.3)	
Coronary heart disease, *n* (%)					<0.001
Yes	873 (3.3)	388 (3.5)	167 (2.4)	318 (3.7)	
No	25,600 (96.7)	10,590 (96.5)	6,657 (97.6)	8,353 (96.3)	
Stroke, *n* (%)					<0.001
Yes	601 (2.3)	221 (2)	118 (1.7)	262 (3)	
No	25,872 (97.7)	10,757 (98)	6,706 (98.3)	8,409 (97)	
Liver disease, *n* (%)					0.003
Yes	859 (3.2)	335 (3.1)	197 (2.9)	327 (3.8)	
No	25,614 (96.8)	10,643 (96.9)	6,627 (97.1)	8,344 (96.2)	
Alcohol Consumption Frequency (day), Median (IQR)	2.0 (1.0, 4.0)	2.0 (1.0, 5.0)	2.0 (1.0, 3.0)	3.0 (2.0, 6.0)	<0.001
Alcohol consumption (drinks per day), Median (IQR)	2.0 (1.0, 3.0)	2.0 (2.0, 4.0)	2.0 (1.0, 3.0)	1.0 (1.0, 3.0)	<0.001
Light, *n* (%)	12,963 (49.0)	4,641 (42.3)	3,178 (46.6)	5,144 (59.3)	
Moderate, *n* (%)	5,758 (21.8)	2,495 (22.7)	1,631 (23.9)	1,632 (18.8)	
Heavy, *n* (%)	7,752 (29.3)	3,842 (35)	2,015 (29.5)	1,895 (21.9)	

OR: odds ratio; CI: confidence interval; IQR: interquartile range. BMI ^a^, body mass index*;* GED *^b^,* general educational development; DKD *^c^*, diabetic kidney disease; HTN *^d^,* hypertension.

### The relationship between alcohol consumption frequency and DKD

#### Univariate analysis

Univariate analysis identified correlations between DKD and age, sex, race, educational level, BMI, smoking status, frequency of alcohol consumption, hypertension, heart failure, coronary artery disease, stroke, and liver disease (Supplementary Table 3).

#### Multivariable analysis

Alcohol consumption was associated with diabetic kidney disease (DKD) (Table 2). After adjusting for confounding factors, each additional standard drink per day was associated with a 3% increase in the odds of DKD (OR, 1.03; 95% CI, 1.00–1.05; *p* = 0.017). Among heavy drinkers, each additional standard drink per day was associated with a 23% increase in the odds of DKD (OR, 1.23; 95% CI, 1.04–1.46; *p* = 0.016).

**Table 2.** Association between drinking patterns and diabetic kidney disease in multivariable regression model.

Variable	Total	Event%	Crude Model		Model **I**		Model **II**		Model **III**	
			OR 95%CI	*P-value*	OR 95%CI	*P-value*	OR 95%CI	*P-value*	OR 95%CI	*P-value*
Alcohol Consumption (drinks per day)	26,473	1,101 (4.2)	0.97 (0.94–0.99)	0.008	1.04 (1.01–1.06)	**0.001**	1.03 (1.01–1.05)	**0.01**	1.03 (1–1.05)	**0.017**
Light, n (%)	12,963	655 (5.1)	1 (Ref)		1 (Ref)		1 (Ref)		1 (Ref)	
Moderate, n (%)	5,758	178 (3.1)	0.6 (0.51–0.71)	<0.001	0.95 (0.79–1.13)	0.535	0.96 (0.8–1.15)	0.651	0.97 (0.81–1.17)	0.775
Heavy, n (%)	7,752	268 (3.5)	0.67 (0.58–0.78)	**<0.001**	1.32 (1.12–1.55)	**0.001**	1.25 (1.06–1.48)	**0.008**	1.23 (1.04–1.46)	**0.016**
Alcohol Consumption Frequency										
Week	10,978	379 (3.5)	1.05 (1–1.1)	0.043	0.95 (0.9–0.99)	0.029	0.97 (0.92–1.02)	0.208	0.96 (0.91–1.01)	0.095
1–2 days [24]	5,763	197 (3.4)	1 (Ref)		1 (Ref)		1 (Ref)		1 (Ref)	
3–4 days	2,463	63 (2.6)	0.74 (0.56–0.99)	**0.042**	0.65 (0.48–0.87)	**0.004**	0.69 (0.51–0.93)	**0.015**	0.67 (0.49–0.91)	**0.01**
5–7 days	2,752	119 (4.3)	1.28 (1.01–1.61)	0.039	0.77 (0.6–0.98)	0.035	0.87 (0.67–1.12)	0.269	0.83 (0.64–1.07)	0.143
Month	6,824	239 (3.5)	0.94 (0.88–1.01)	0.073	0.93 (0.87–0.99)	**0.027**	0.93 (0.87–0.99)	**0.033**	0.93 (0.87–0.99)	**0.025**
1 day	2,729	116 (4.3)	1 (Ref)		1 (Ref)		1 (Ref)		1 (Ref)	
2–5 days	3,492	105 (3)	0.7 (0.53–0.91)	**0.009**	0.73 (0.55–0.96)	**0.027**	0.74 (0.56–0.99)	**0.04**	0.75 (0.56–0.99)	**0.045**
≥5 days	603	18 (3)	0.69 (0.42–1.15)	0.154	0.61 (0.36–1.03)	0.066	0.64 (0.38–1.09)	0.101	0.63 (0.37–1.08)	0.095
Year	8,671	483 (5.6)	0.998 (0.995–1.002)	0.334	0.998 (0.994–1.001)	0.114	0.998 (0.995–1.001)	0.259	0.998 (0.995–1.001)	0.282
<3 days	3,236	224 (6.9)	1 (Ref)		1 (Ref)		1 (Ref)		1 (Ref)	
3–126 days	5,295	253 (4.8)	0.67 (0.56–0.81)	**<0.001**	0.68 (0.56–0.82)	**<0.001**	0.7 (0.58–0.86)	**<0.001**	0.71 (0.58–0.86)	**0.001**
≥126 days	140	6 (4.3)	0.6 (0.26–1.38)	0.23	0.44 (0.19–1.02)	0.057	0.5 (0.21–1.18)	0.112	0.52 (0.22–1.23)	0.136

OR: odds ratio; CI, confidence interval; Ref: reference.

Crude model adjusted for: none.

Model I adjusted for: age, race, sex.

Model II adjusted for: Model I + education level, smoking status, body mass index, and milk consumption.

Model III adjusted for: Model II+ heart failure, coronary heart disease, stroke, liver disease, and hypertension.

The frequency of alcohol consumption was also associated with DKD. Monthly alcohol consumption frequency was inversely associated with DKD (OR, 0.93; 95% CI, 0.87–0.99; *p* = 0.025). For weekly alcohol consumption, participants consuming alcohol 3–4 days per week had an adjusted odds ratio of 0.67 (95% CI, 0.49–0.91; *p* = 0.01) compared with those consuming alcohol 1–2 days per week. For yearly alcohol consumption, participants consuming alcohol 3–126 days per year had an adjusted odds ratio of 0.71 (95% CI, 0.58–0.86; *p* = 0.001) compared with those consuming alcohol <3 days per year.

#### Threshold analysis

Threshold analysis indicated that participants with a weekly alcohol consumption frequency of <4 days had an odds ratio of 0.763 (95% CI, 0.662–0.879; *p* = 0.0002) for DKD (Supplementary Table 4). No significant association was observed for weekly alcohol consumption frequency ≥4 days (Supplementary Table 4). Similarly, participants with an annual alcohol consumption frequency of <126 days had an odds ratio of 0.985 (95% CI, 0.970–0.999; *p* = 0.038) for DKD.

Restricted cubic spline analysis demonstrated an L-shaped association between weekly and yearly alcohol consumption frequency and DKD (nonlinear *p* = 0.003 for weekly frequency, nonlinear *p* = 0.004 for yearly frequency) (Supplementary Figures 1 and 2; Supplementary Table 4).

### Subgroup analysis

Stratified analyses were conducted within subgroups defined by age, sex, body mass index (BMI), and race to evaluate the potential effect of alcohol consumption volume and frequency on DKD. Interactions for alcohol consumption volume were observed in race subgroups. Subgroup analysis revealed differences in the association between alcohol consumption volume and DKD across racial groups (Supplementary Figures 3–6).

### Sensitivity analysis

We conducted sensitivity analyses to assess the robustness of our findings regarding missing data. First, we compared the characteristics of participants excluded due to missing data with those included in the analysis to identify potential systematic differences (Supplementary Table 1). We then used multiple imputation (MICE) to impute missing values for key confounders, including BMI, blood pressure, and smoking status. The results before and after imputation were consistent (Supplementary Table 2), indicating that imputation did not significantly affect the conclusions. These analyses confirmed the robustness of our results.

## Discussion

This large cross-sectional study gathered data from 26,473 participants aged ≥ 20 who participated in the NHANES from 1999 to 2016. A statistically significant association between drinking patterns and the risk of DKD was consistently observed in our analysis. Importantly, our findings indicated a dose-response relationship, demonstrating that alcohol consumption frequency was correlated with DKD in an L-shaped non-linear manner (*P* for nonlinearity < 0.05). The strength of this association decreased as the alcohol consumption frequency increased, indicating that the protective effect against DKD weakened with higher levels of alcohol intake.

Additionally, we found a statistically significant interaction between alcohol consumption and race in predicting DKD (*p* < 0.05). Subsequent subgroup analyses within the age, sex, and BMI groups did not reveal any significant interactions among the subgroups. These findings have significant implications for the management of DKD across diverse populations, highlighting the need for further investigation of the differences among various demographic groups. It is crucial to underscore the clinical relevance of these findings in influencing the current strategies for DKD management. The observed associations and interactions not only enhance our understanding of the link between drinking patterns and DKD but also underscore the need to consider population-specific factors in disease management strategies. Further research is needed to elucidate the underlying mechanisms that drive these associations and explore tailored interventions for different population groups.

Our study investigated the complex and nonlinear associations between drinking patterns and DKD by analyzing data from 26,473 participants aged ≥ 20 years in the 1999–2016 NHANES. Our findings are similar to those of previous studies exploring the relationship between alcohol consumption and the risk of diabetic kidney disease (DKD). For example, van der Heide et al. [8] described a nonlinear relationship between alcohol consumption and DKD risk, characterized by a J-shaped curve. In contrast, we observed an L-shaped relationship, in which the risk of DKD decreased with increased drinking frequency before plateauing.

These divergent trends beyond the threshold may stem from differences in sample sizes, study populations, and methods used to define alcohol consumption behavior and DKD outcomes. Our study included 26,473 participants aged ≥ 20 years, whereas van der Heide et al. [8] examined 2,318 individuals aged ≥ 40 years. Additionally, we defined proteinuria using the albumin-to-creatinine ratio (ACR), whereas van der Heide et al. employed 24-h urinary albumin excretion (UAE). Variations in these parameters can influence the observed relationships.

Roy et al. [15] also identified an association between a lack of moderate alcohol consumption and an elevated risk of DKD. This aligns with our observation that heavy alcohol consumption is associated with a higher risk of DKD. This effect may be related to the potential of moderate alcohol consumption to enhance insulin sensitivity, reduce insulin resistance, and subsequently decrease the risk of vascular dysfunction and kidney disease [8,16–20].

In the subgroup analysis, our findings should be interpreted in the context of a potential selection bias. The forest plot suggests a significant protective effect of alcohol on DKD among participants aged 65 and older, which may reflect a selection bias where relatively healthier older individuals are more likely to consume alcohol. This ‘healthy user’ effect is commonly observed in aging populations [21–23], where individuals’ health status influences their drinking behaviors. Therefore, although our results demonstrate this association, caution is warranted in their interpretation, and further studies are needed to explore the potential confounding factors underlying this relationship. Although the P-values for the interactions between alcohol consumption and race were < 0.05, these findings may not have substantial clinical implications because of the potential for multiple testing bias and the consistent directionality of associations (Supplementary Figure 1).

Prior studies have investigated the relationship between drinking patterns and the prevalence of DKD within the framework of the Prevention of Renal and Vascular End-Stage Disease (PREVEND) study [16], a large prospective population-based cohort. This study included a substantial number of outcome cases and extended the follow-up period, providing sufficient statistical power for detailed subgroup analyses. The baseline data included a wide range of lifestyle exposures, including other dietary factors, allowing for the adjustment of potential confounding variables. This enables the execution of a comprehensive set of sensitivity analyses, enhances the interpretability of the results, and validates key findings, which support the results of our study.

Several limitations must be considered when interpreting the results of this study. First, the observational nature of the study limits its ability to establish definitive causal inferences. Although a correlation between drinking patterns and the risk of diabetic kidney disease (DKD) has been identified, a direct causal link cannot be established. Longitudinal studies with extended follow-up periods are necessary to evaluate the long-term impact of alcohol consumption on DKD development and to clarify the causal relationships. Second, although our multivariable models are robust, the potential remains for residual confounding by unmeasured or unknown variables, such as physical activity or household income. These unaccounted factors could influence the observed associations, potentially leading to either an over- or underestimation of the true relationships. However, the control for several critical confounders suggests that the impact of any residual confounding is likely minimal. Third, the study was conducted using data from a US population, which may limit the generalizability of the findings to other populations with different drinking habits and DKD risk profiles. Future research should include diverse populations to validate these findings and explore the cultural, genetic, and environmental factors that may modulate the relationship between alcohol consumption and DKD risk. A further limitation of our study is the observed differences between excluded and included participants, particularly due to nonrandom missing data. Although these differences were significant, they did not have any notable clinical implications. Nonetheless, these discrepancies may have affected the generalizability of our findings. Future research should address these issues by exploring how nonrandom missing data might influence the conclusions of similar studies, and caution should be exercised when interpreting our findings. Future prospective studies are necessary to confirm these results and further our understanding of the relationship between alcohol consumption patterns and DKD.

In conclusion, this study highlighted the impact of appropriate drinking patterns on the prevalence of DKD, independent of confounders. A nonlinear L-shaped association was observed between drinking frequency and DKD, indicating varying levels of risk at different drinking frequencies, with inflection points at 4 days (weekly drinking frequency) and 126 days (yearly drinking frequency). This association is relevant to clinicians when developing management strategies for DKD. However, the results of the present study require further validation.

## Conclusions

This study suggests an association between moderate alcohol consumption frequency and a reduced risk of DKD. However, causality is not established, and any potential benefits must be balanced against the potentially harmful effects of alcohol, such as an increased risk of hypoglycemia, liver complications, and certain cancers [4].

## Supplementary Material

Supplementary_information new.docx

## Data Availability

All data in the article can be obtained from NHANES database (https://www.cdc.gov/nchs/nhanes/index.htm).
