# Supplementary material for: Association between drinking patterns and diabetic kidney disease in United States adults: a cross-sectional study based on data from NHANES 1999–2016
Source: Ren Fail. 2025 Jan 22;47(1):2454970. doi: 10.1080/0886022X.2025.2454970 (PMC11755733; doi:10.1080/0886022X.2025.2454970)
Supplement: Supplementary_information new.docx [file IRNF_A_2454970_SM4209.docx]

Supplementary information

Supplementary Table 1. Comparison of basic characteristics between excluded and included populations.

| Variables | Age ≥20 years  Total Number | Included | Excluded | p |
| --- | --- | --- | --- | --- |
|  | (n = 49,512) | (n = 26,473) | (n = 23,039) |  |
| Age, Mean ± SD | 49.7 ± 18.5 | 46.6 ± 17.3 | 53.3 ± 19.1 | < 0.001 |
| Sex, n (%) |  |  |  | < 0.001 |
| Male | 23,771 (48.0) | 14,209 (53.7) | 9,562 (41.5) |  |
| Female | 25,741 (52.0) | 12,264 (46.3) | 13,477 (58.5) |  |
| Race, n (%) |  |  |  | < 0.001 |
| Mexican American | 8,858 (17.9) | 4,631 (17.5) | 4,227 (18.3) |  |
| Other Hispanic | 4,005 (8.1) | 2,068 (7.8) | 1,937 (8.4) |  |
| Non-Hispanic White | 22,369 (45.2) | 13,054 (49.3) | 9,315 (40.4) |  |
| Non-Hispanic Black | 10,218 (20.6) | 4,873 (18.4) | 5,345 (23.2) |  |
| Other Race | 4,062 (8.2) | 1,847 (7) | 2,215 (9.6) |  |
| Education, n (%) |  |  |  | < 0.001 |
| Below high school | 14,023 (28.3) | 5,825 (22) | 8,198 (35.6) |  |
| High school grad/GED ^b^ /Equivalent | 11,408 (23.0) | 5,957 (22.5) | 5,451 (23.7) |  |
| Above high school | 23,974 (48.4) | 14,675 (55.4) | 9,299 (40.3) |  |
| BMI, Mean ± SD | 28.8 ± 6.7 | 28.6 ± 6.5 | 29.2 ± 7.0 | < 0.001 |
| Milk, n (%) |  |  |  | < 0.001 |
| Never | 8,189 (16.5) | 4,333 (16.4) | 3,856 (16.8) |  |
| Rarely (less than once a week) | 7,455 (15.1) | 4,180 (15.8) | 3,275 (14.2) |  |
| Sometimes (once a week or more) | 13,421 (27.1) | 7,554 (28.5) | 5,867 (25.5) |  |
| Often (once a day or more) | 20,193 (40.8) | 10,302 (38.9) | 9,891 (43) |  |
| Varied | 234 (0.5) | 103 (0.4) | 131 (0.6) |  |
| Hypertension, n (%) |  |  |  | < 0.001 |
| No | 35,502 (71.9) | 20,087 (76.1) | 15,415 (67.1) |  |
| Yes | 13,855 (28.1) | 6,308 (23.9) | 7,547 (32.9) |  |
| Diabetic Nephropathy, n (%) |  |  |  | < 0.001 |
| No | 46,255 (93.4) | 25,372 (95.8) | 20,883 (90.6) |  |
| Yes | 3,257 (6.6) | 1,101 (4.2) | 2,156 (9.4) |  |
| Heart Failure, n (%) |  |  |  | < 0.001 |
| Yes | 1,705 (3.4) | 550 (2.1) | 1,155 (5) |  |
| No | 47,804 (96.6) | 25,922 (97.9) | 21,882 (95) |  |
| Coronary heart disease, n (%) |  |  |  | < 0.001 |
| Yes | 2,086 (4.2) | 873 (3.3) | 1,213 (5.3) |  |
| No | 47,423 (95.8) | 25,599 (96.7) | 21,824 (94.7) |  |
| Stroke, n (%) |  |  |  | < 0.001 |
| Yes | 1,924 (3.9) | 601 (2.3) | 1,323 (5.7) |  |
| No | 47,585 (96.1) | 25,871 (97.7) | 21,714 (94.3) |  |
| Liver disease, n (%) |  |  |  | < 0.001 |
| Yes | 1,771 (3.6) | 858 (3.2) | 913 (4) |  |
| No | 47,738 (96.4) | 25,614 (96.8) | 22,124 (96) |  |

Supplementary Table 2: Comparison of data before and after multiple imputation

| Variable | Pre-multiple imputation data | *P-value* | post-multiple imputation data | *P-value* |
| --- | --- | --- | --- | --- |
|  | OR 95%CI |  | OR 95%CI |  |
| Age | 1.06 (1.05–1.06) | <0.001 | 1.06 (1.05–1.06) | <0.001 |
| Sex, Female Vs Male | 0.64 (0.56–0.72) | <0.001 | 0.6 (0.52–0.69) | <0.001 |
| Race |  |  |  |  |
| Mexican American Vs Non-Hispanic White | 1.38 (1.17–1.63) | <0.001 | 1.29 (1.09–1.53) | 0.003 |
| Other Hispanic Vs Non-Hispanic White | 1.36 (1.08–1.71) | 0.008 | 1.32 (1.05–1.67) | 0.017 |
| Non-Hispanic Black Vs Non-Hispanic White | 1.91 (1.64–2.22) | <0.001 | 1.61 (1.38–1.87) | <0.001 |
| Other Race Vs Non-Hispanic White | 0.97 (0.74–1.28) | 0.838 | 1.13 (0.85–1.48) | 0.401 |
| BMI | 1.07 (1.07–1.08) | <0.001 | 1.08 (1.07–1.09) | <0.001 |
| Smoking |  |  |  |  |
| Sometimes Vs Everyday | 0.93 (0.7–1.24) | 0.63 | 1.06 (0.76–1.49) | 0.739 |
| Never Vs Everyday | 1.7 (1.47–1.95) | <0.001 | 0.85 (0.72–1) | 0.056 |
| Milk |  |  |  |  |
| Rarely (less than once a week) Vs Never | 0.93 (0.75–1.15) | 0.489 | 1.06 (0.84–1.33) | 0.611 |
| Sometimes (once a week or more) Vs Never | 1.01 (0.84–1.21) | 0.932 | 1.11 (0.91–1.35) | 0.306 |
| Often (once a day or more) Vs Never | 0.95 (0.8–1.14) | 0.593 | 0.99 (0.81–1.19) | 0.889 |
| Varied Vs Never | 1.14 (0.46–2.84) | 0.772 | 1.12 (0.43–2.91) | 0.82 |
| Education |  |  |  |  |
| High school grad/GED ^b^ /Equivalent Vs Below high school | 0.7 (0.59–0.82) | <0.001 | 0.87 (0.73–1.05) | 0.143 |
| Above high school Vs Below high school | 0.56 (0.49–0.65) | <0.001 | 0.85 (0.73–1) | 0.057 |
| HTN *^c^* Yes Vs No | 5.37 (4.74–6.08) | <0.001 | 2.17 (1.89–2.49) | <0.001 |
| Heart Failure No Vs Yes | 0.12 (0.1–0.15) | <0.001 | 0.45 (0.36–0.57) | <0.001 |
| Coronary heart disease No Vs Yes | 0.19 (0.16–0.23) | <0.001 | 0.67 (0.53–0.83) | <0.001 |
| Stroke No Vs Yes | 0.19 (0.16–0.24) | <0.001 | 0.55 (0.43–0.7) | <0.001 |
| Liver disease No Vs Yes | 0.55 (0.42–0.72) | <0.001 | 0.8 (0.6–1.07) | 0.134 |
| Alcohol Consumption Frequency |  |  |  |  |
| Monthly Vs Weekly | 1.02 (0.86–1.2) | 0.859 | 1.2 (1–1.43) | 0.045 |
| Yearly Vs Weekly | 1.65 (1.44–1.89) | <0.001 | 1.45 (1.24–1.7) | <0.001 |
| Alcohol Consumption Frequency (day), Median (IQR) | 1 (1–1) | 0.831 | 1 (0.99–1) | 0.214 |
| Alcohol consumption  (drinks per day), Median (IQR) | 0.97 (0.94–0.99) | 0.008 | 1.03 (1.01–1.06) | 0.003 |

Supplementary Table 3: The results of univariate analysis

| Variable | OR 95%CI | *P-value* |
| --- | --- | --- |
| Age | 1.06 (1.05–1.06) | <0.001 |
| Sex, Female Vs Male | 0.64 (0.56–0.72) | <0.001 |
| Race |  |  |
| Mexican American Vs Non-Hispanic White | 1.38 (1.17–1.63) | <0.001 |
| Other Hispanic Vs Non-Hispanic White | 1.36 (1.08–1.71) | 0.008 |
| Non-Hispanic Black Vs Non-Hispanic White | 1.91 (1.64–2.22) | <0.001 |
| Other Race Vs Non-Hispanic White | 0.97 (0.74–1.28) | 0.838 |
| BMI ^a^ | 1.07 (1.07–1.08) | <0.001 |
| Smoking |  |  |
| Sometimes Vs Everyday | 0.88 (0.69–1.13) | 0.324 |
| Never Vs Everyday | 1.75 (1.53–2) | <0.001 |
| Education |  |  |
| High school grad/GED ^b^ /Equivalent Vs Below high school | 0.7 (0.59–0.82) | <0.001 |
| Above high school Vs Below high school | 0.56 (0.49–0.65) | <0.001 |
| Alcohol Consumption Frequency |  |  |
| Monthly Vs Weekly | 1.02 (0.86–1.2) | 0.859 |
| Yearly Vs Weekly | 1.65 (1.44–1.89) | <0.001 |
| Alcohol Consumption Frequency(day) | 1 (1–1) | 0.831 |
| Alcohol consumption  (drinks per day) | 0.97 (0.94–0.99) | 0.008 |
| HTN *^c^* Yes Vs No | 5.37 (4.74–6.08) | <0.001 |
| SBP | 1.04 (1.03–1.04) | <0.001 |
| DBP | 0.99 (0.99–1) | 0.012 |
| Milk |  |  |
| Rarely (less than once a week) Vs Never | 0.93 (0.75–1.15) | 0.489 |
| Sometimes (once a week or more) Vs Never | 1.01 (0.84–1.21) | 0.932 |
| Often (once a day or more) Vs Never | 0.95 (0.8–1.14) | 0.592 |
| Varied Vs Never | 1.14 (0.46–2.84) | 0.772 |
| Heart Failure No Vs Yes | 0.12 (0.1–0.15) | <0.001 |
| Coronary heart disease No Vs Yes | 0.19 (0.16–0.23) | <0.001 |
| Stroke No Vs Yes | 0.19 (0.16–0.24) | <0.001 |
| Liver disease No Vs Yes | 0.55 (0.42–0.72) | <0.001 |
| ACEI/ARB Yes Vs No | 2.55 (2.05–3.17) | <0.001 |
| HbA1C | 2.52 (2.41–2.63) | <0.001 |
| LDL | 0.73 (0.66–0.81) | <0.001 |

OR, odds ratio; CI, conﬁdence interval; BMI ^a^*,* body mass index*;* GED *^b^,* general educational development; HTN *^c^,* hypertension

Supplementary Table 4. Analysis of the threshold effect in the relationship between alcohol consumption frequency and DN.

| Alcohol Consumption Frequency | Adjust model | |
| --- | --- | --- |
|  | OR (95%CI) | *P value* |
| Week |  |  |
| <4 days | 0.763 (0.662–0.879) | 0.0002 |
| ≥4 days | 1.13 (0.883–1.445) | 0.3316 |
| Likelihood Ratio test |  | 0.003 |
| Year |  |  |
| <126 days | 0.985 (0.97–0.999) | 0.038 |
| ≥126 days | 1.476 (0–Inf) | 0.9995 |
| Likelihood Ratio test |  | 0.007 |

OR, odds ratio; CI, conﬁdence interval. Adjusted for sociodemographic factors (age, sex, race, and education level), smoking status, body mass index, milk consumption, hypertension, heart failure, coronary heart disease, stroke, and liver disease. Only 99.9% of the data are displayed.


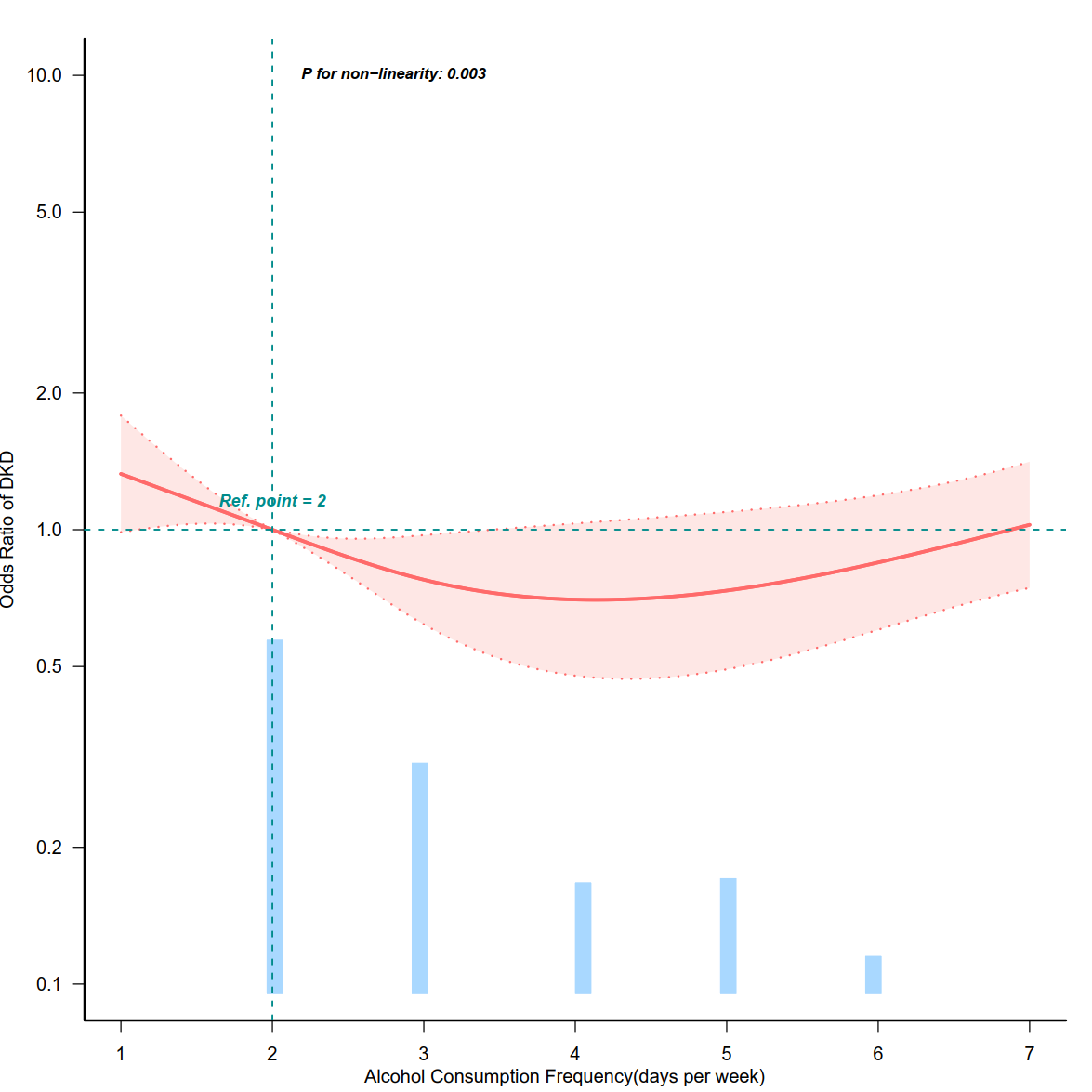


Supplementary Figure 1. Association between weekly frequency of alcohol consumption and odd ratio of diabetic nephropathy. Solid and dashed lines represent the predicted value and 95% confidence intervals. They were adjusted for sociodemographic factors (age, sex, race, education level), smoking status, body mass index, milk consumption, hypertension, heart failure, coronary heart disease, stroke, and liver disease. Chart shows 99.9% of the data.


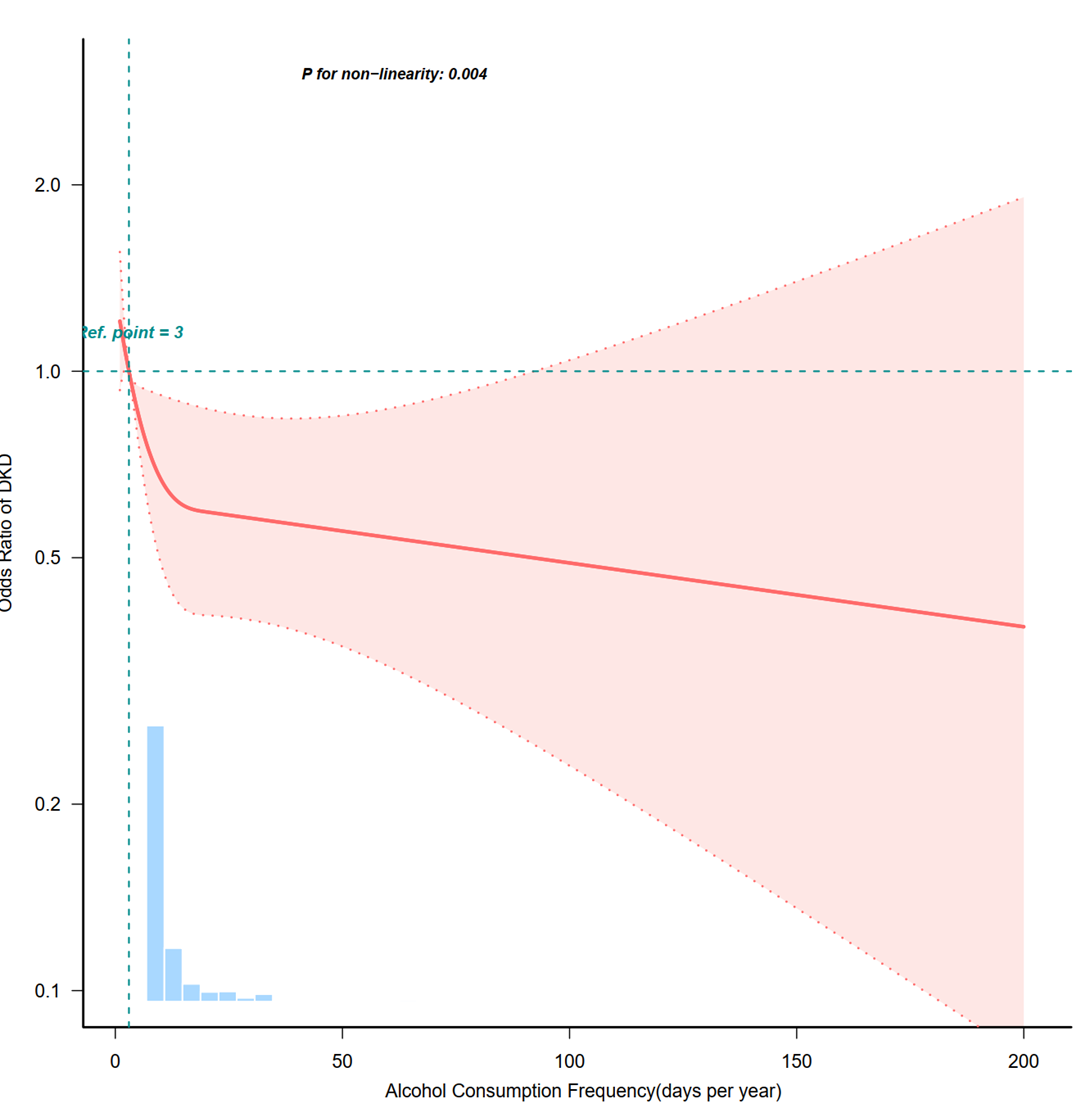


Supplementary Figure 2. Association between yearly frequency of alcohol consumption and odds ratio of diabetic nephropathy. Solid and dashed lines represent the predicted value and 95% confidence intervals. They were adjusted for sociodemographic factors (age, sex, race, education level), smoking status, body mass index, milk consumption, hypertension, heart failure, coronary heart disease, stroke, and liver disease. Chart shows 99% of the data.


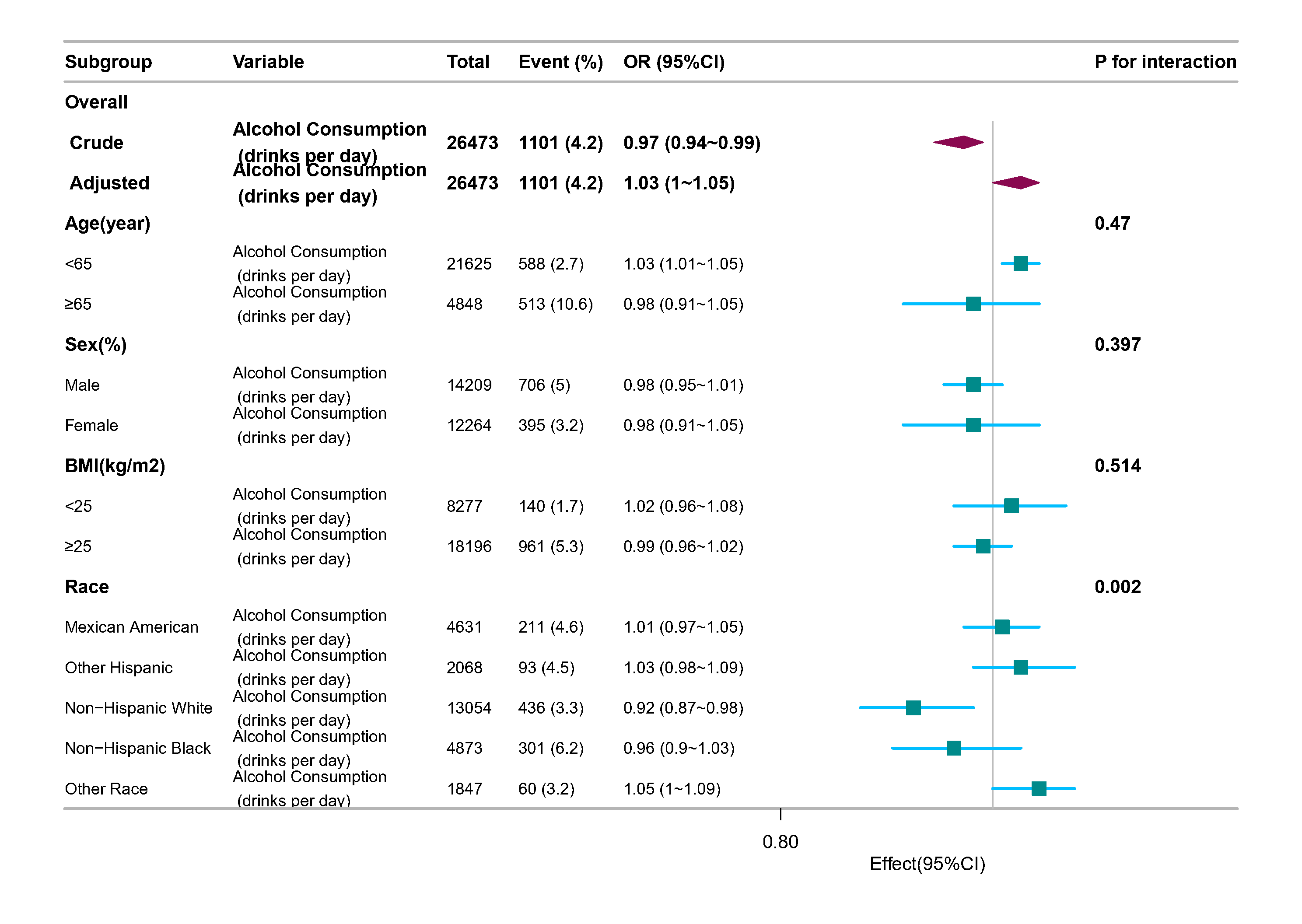
Supplementary Figure 3. Forest plot of the effect of alcohol consumption on the outcome of diabetic nephropathy in subgroups adjusted for smoking history, education level, milk consumption, hypertension, coronary heart disease, heart failure, stroke, and history of liver disease.


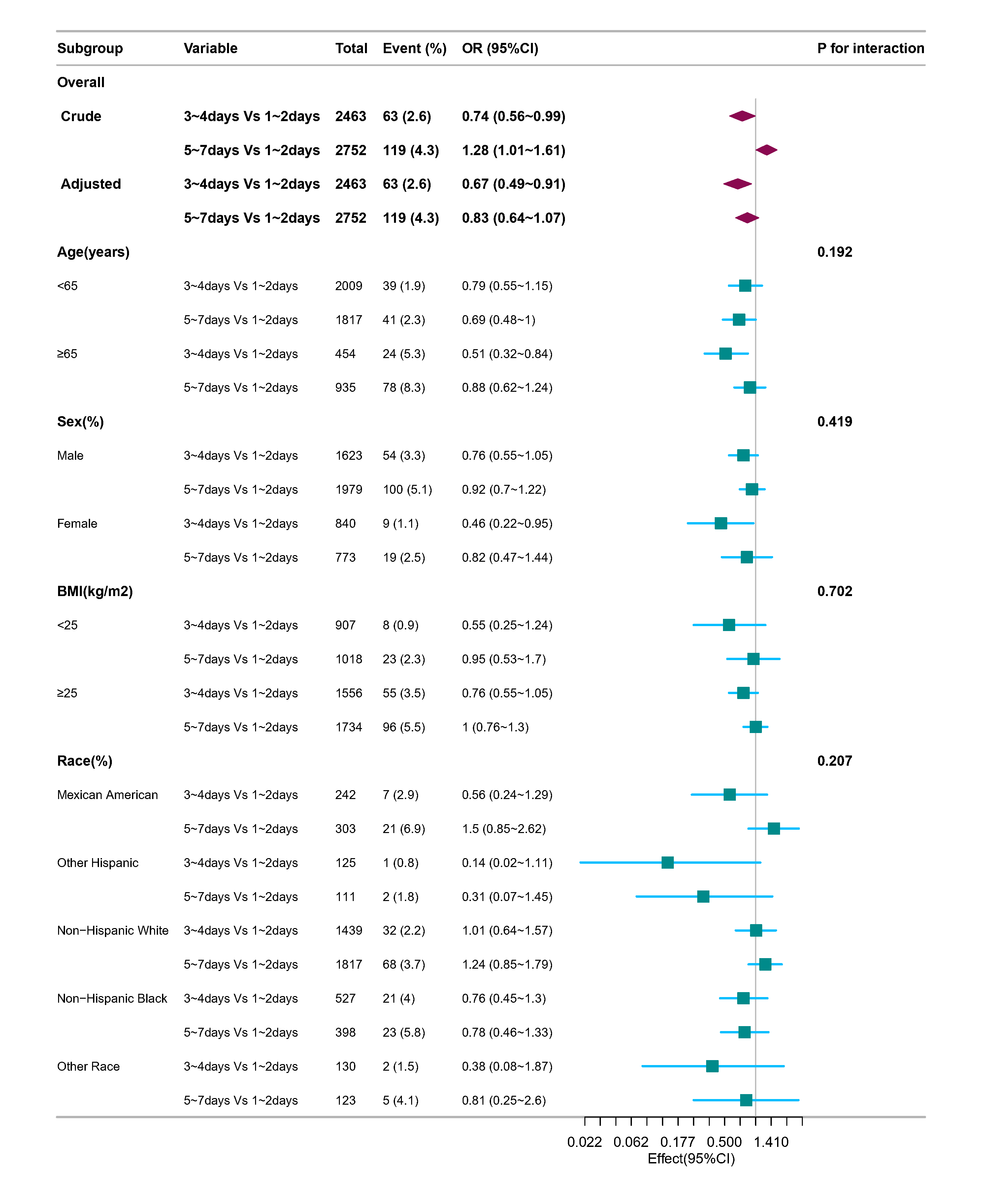
 Supplementary Figure 4. Forest plot of the effect of alcohol consumption frequency (weekly) on the outcome of diabetic nephropathy in subgroups adjusted for smoking history, education level, milk consumption, hypertension, coronary heart disease, heart failure, stroke, and history of liver disease.


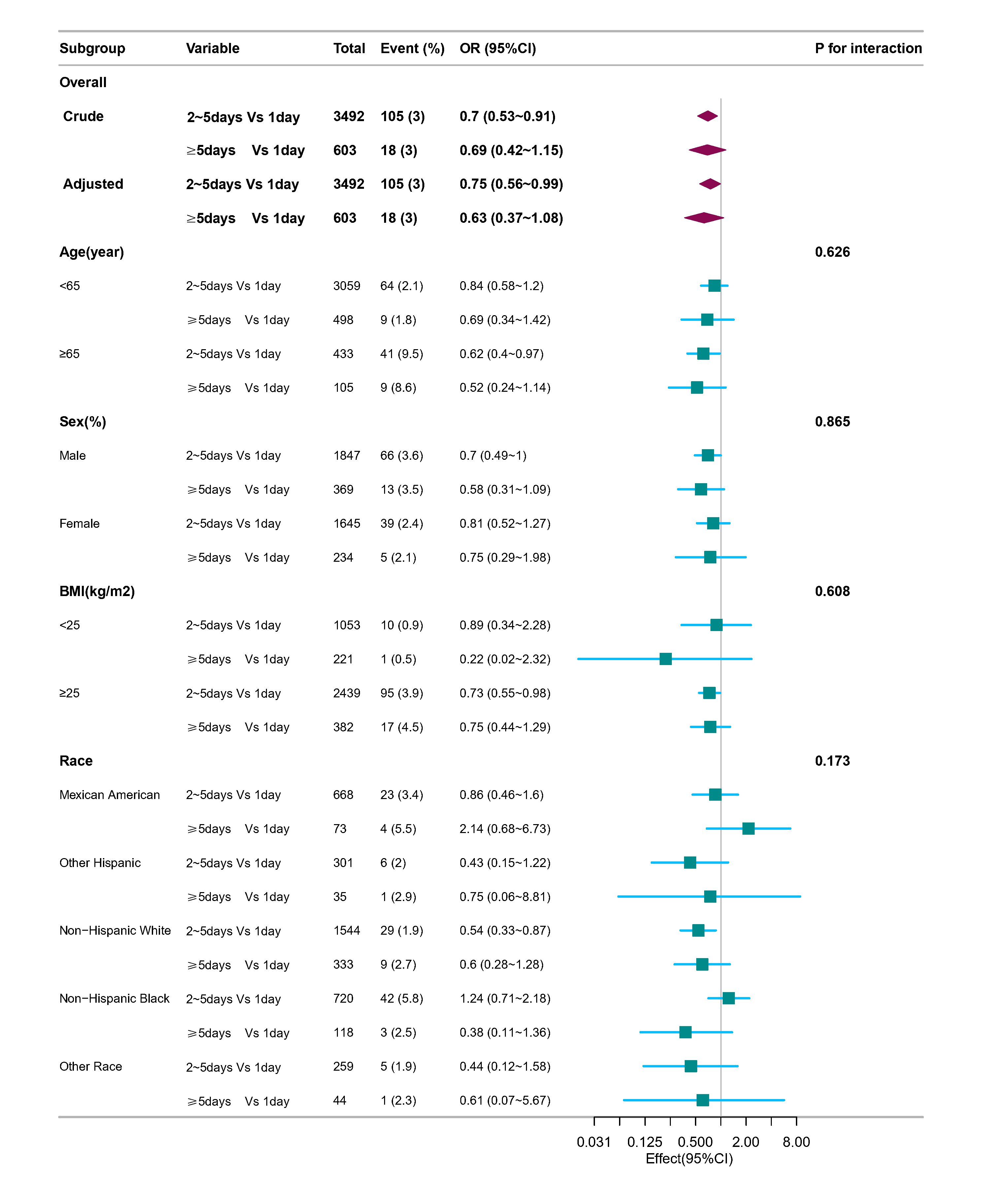


Supplementary Figure 5. Forest plot of the effect of alcohol consumption frequency (monthly) on the outcome of diabetic nephropathy in subgroups adjusted for smoking history, education level, milk consumption, hypertension, coronary heart disease, heart failure, stroke, and history of liver disease.


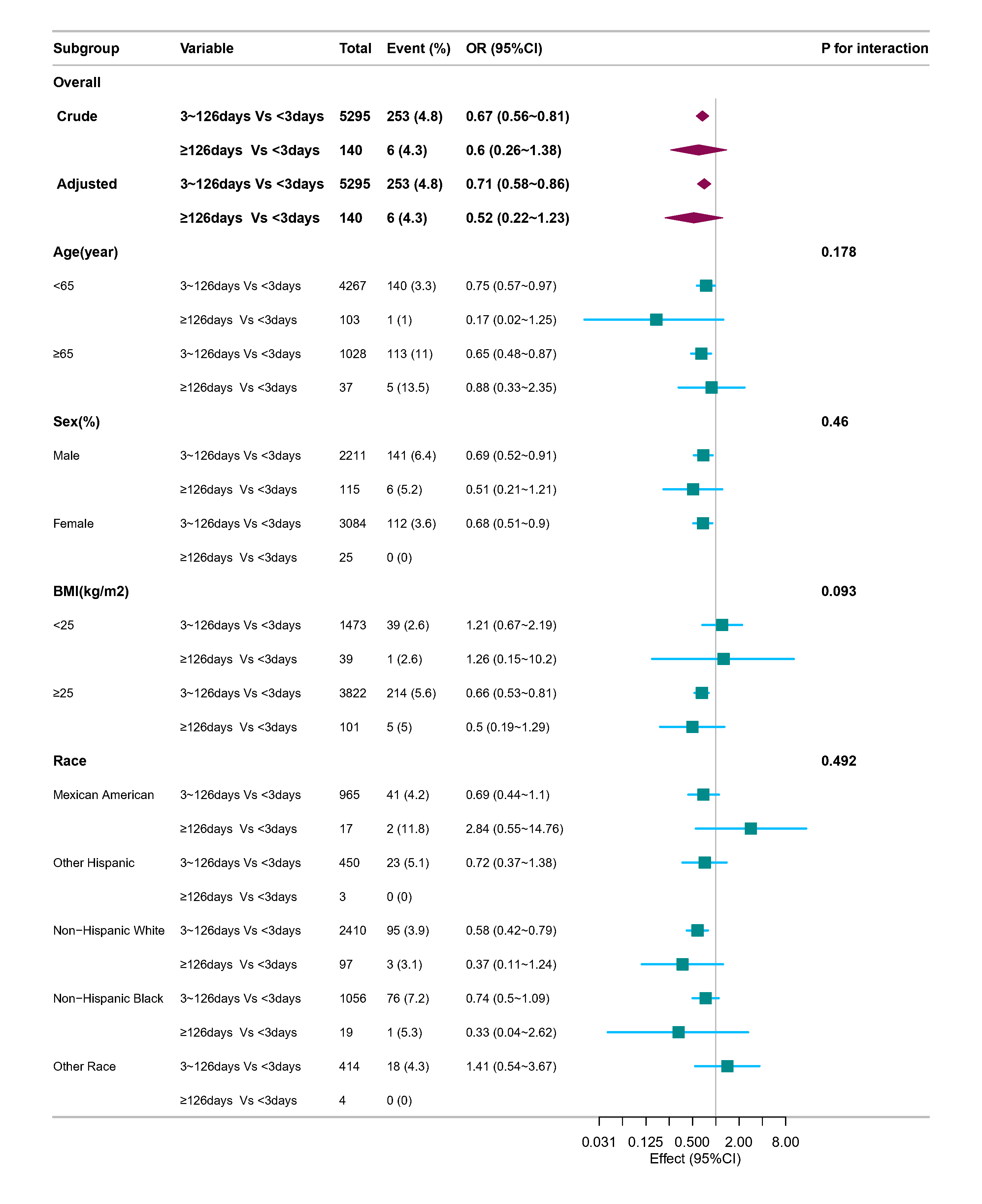
Supplementary Figure 6. Forest plot of the effect of alcohol consumption frequency (yearly) on the outcome of diabetic nephropathy in subgroups adjusted for smoking history, education level, milk consumption, hypertension, coronary heart disease, heart failure, stroke, and history of liver disease.
